# Co-roosting relationships are consistent across years in a bat maternity group

**DOI:** 10.1038/s41598-023-50191-4

**Published:** 2024-01-16

**Authors:** Julia Sunga, Jessica Humber, Hugh G. Broders

**Affiliations:** 1 Department of Fisheries, Forestry and Agriculture, Government of Newfoundland and Labrador, 192 Wheeler’s Road, PO Box 2007, Corner Brook, NL A2H 7S1 Canada; 2 Department of Biology, Saint Mary’s University, Halifax, NS B3H 3C3 Canada; 3 Present Address: Department of Biology, University of Waterloo, 200 University Ave W, Waterloo, ON N2L 3G1 Canada

**Keywords:** Behavioural ecology, Population dynamics

## Abstract

Long-lived, group living animals have the potential to form multiyear relationships. In some temperate bat species, maternity groups break apart and rejoin both daily, as females depart to forage and select day roosts to use, and annually, as bats leave for and return from hibernation. Here, we investigated whether bats have persistent social preferences by testing whether relationships between dyads in a focal year could be predicted by previous years. We also hypothesized that experience influences social preferences and predicted that an individual’s age would influence its network position, while familiarity with bats of the same cohort would drive persistent social preferences. We quantified roost co-occurrence in little brown myotis (*Myotis lucifugus*) in Salmonier Nature Park, Newfoundland, Canada both within and among years. We found that roost co-occurrence patterns of previous years still had predictive value even when accounting for potential roost fidelity. However, we found no evidence that cohort familiarity or age explained any of the variation. Overall, we found long-term patterns of association in this temperate bat species that suggest levels of social complexity akin to other large mammal species.

## Introduction

Understanding which factors explain the persistence of group behaviour and social relationships among individuals remains an ongoing challenge in behavioural ecology. The persistence of group behaviour requires that individuals continually interact through mechanisms such as social attraction or aggregation around common resources^1,2^, while long-term social bonds require factors such as spatial memory to return to the same locations^3,4^ and/or social memory to recognize associates over time^5–7^. Long-lived, group living animals have the potential to form long-term social bonds that persist for many years and span generations.

Fission–fusion social systems describe animal groups where one or more individuals break away from and rejoin subgroups in changing conformations over various periods of time^8,9^. As groups break apart and rejoin, there is the opportunity for social relationships to exist at different temporal and spatial scales. Individuals may separate simply between different times of day to forage, or at a much larger temporal and spatial scales, gather during certain times of year such as during the breeding season^10^. Thus, both transient and long-term relationships may exist within an animal group that displays fission–fusion dynamics.

Network analysis is a useful way of visualizing and quantifying the relationships between individuals and its application in animal groups has expanded greatly in recent years^11^. In many applications, networks represent an aggregate of interactions that occur over a finite period of time^12–14^. Comparing time-aggregated networks across intervals may reveal persistent relationships between individuals and possible mechanisms driving the maintenance of group structure. When using time-aggregated networks, one significant challenge is determining the most informative time step length for the species under study or the research question at hand, as these decisions can have profound implications on estimated network structure and strength of ties between individuals^15–17^. Time-aggregated networks are particularly useful when animals only exist together or in a study area during certain times of year separated by long (multi-week or multi-month) periods of absence, either from each other or a particular geographic region.

Throughout the spring and summer, female temperate bats of some species roost in groups, and may use multiple roosts within a maternity area^18,19^. Previously, it has been demonstrated that temperate female bats interact nonrandomly at maternity roost sites suggesting a role of social preferences in shaping maternity groups^20,21^. It has also been shown that not all patterns of co-roosting can be accounted for by roost fidelity, thus further implicating a role of social preferences in shaping temperate bat groups^21^. During the maternity period, females return from hibernation sites, gestate, give birth to their young, then nurse their young for approximately 3-weeks until the pups reach volancy, after which point they depart to swarming and hibernation sites^18,22,23^. It is known that in multiple temperate bat species, at least some females are philopatric^19,24–27^. As individuals enter or leave the maternity sites between maternity periods and return to maternity sites from possibly extended periods apart, or at least away from maternity sites during swarming and hibernation, it is of interest to understand if relationships between bats persist through time.

In long-lived species, social preferences and relationships may also change with age. Temperate bats have been regularly recorded to live over ten years, with a record of a little brown myotis (*Myotis lucifugus*) of at least 34 years old in Saskatchewan, Canada^28^. Exactly how age affects individual habitat use and social preferences is uncertain however, as results vary between taxa. In male African elephants (*Loxodonto africana*), older individuals had more stable network centrality, while younger males were far more variable^29^. Meanwhile in Northern long-eared bats (*Myotis septentrionalis*) there is evidence supporting the idea that juveniles play an important role in maintaining connections across the population^30^ and thus hold a more central position in modelled networks.

In this study we hypothesized that female bats select daily roosts based, at least in part, on social preferences that persist across years. Specifically, we predicted that given their long-lived nature, social relationships between bats in maternity groups will persist across years more often than expected by random chance interactions. As roost fidelity is related to roost co-occurrence patterns, we also predicted that roost association patterns in previous years would be predictive of patterns in the focal year, even when roost use frequencies are accounted for. Subsequently, we hypothesized that persistent social preferences may be informed by experience in the system and familiarity with conspecifics. As such, we predicted that a bat’s age and therefore its experience in a system will influence the social preferences and network position of an individual. Specifically, that older individuals will be more selective of their associates and therefore interact with fewer conspecifics, while younger individuals will have higher centrality as demonstrated by Patriquin et al.^30^. We also predicted that, regardless of age, bats of the same cohort would be most familiar with each other and, as such, roost most frequently with each other.

## Results

### Social association patterns across years

**Figure 1:**
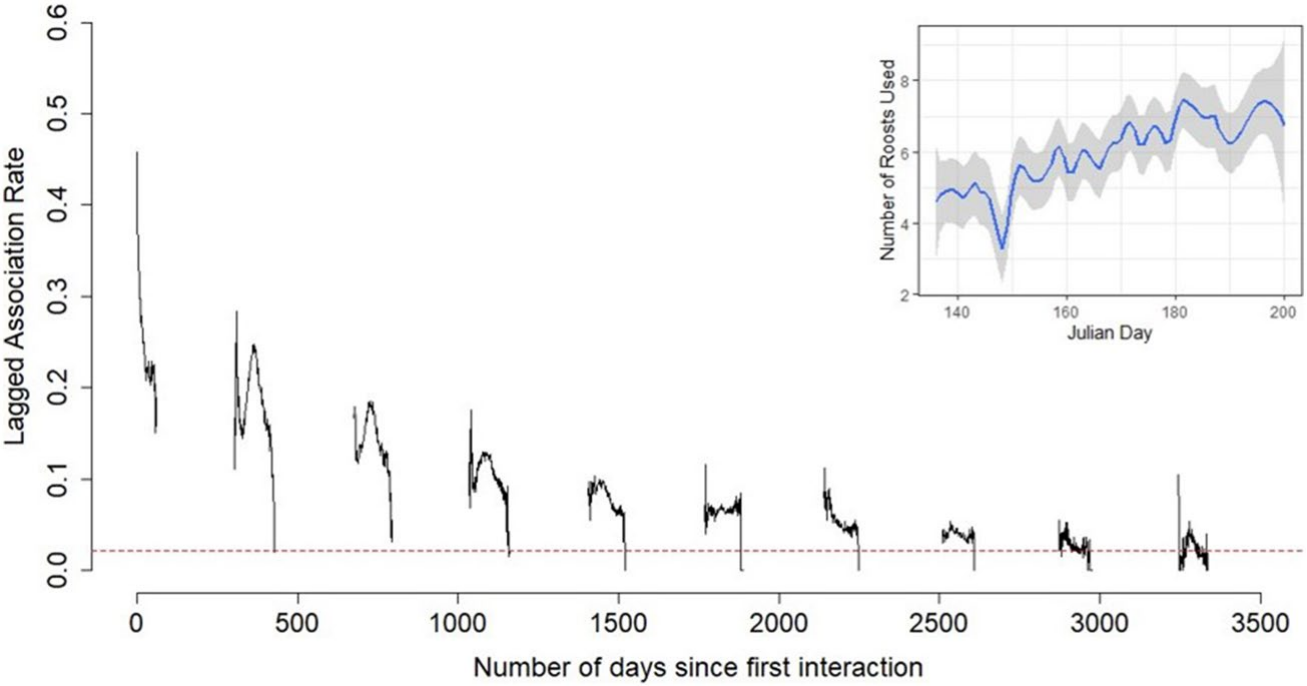
Lagged association rates for female little brown myotis with at least 10 day roost observations during the pregnancy period from 2012 to 2021 in Salmonier Nature Park demonstrating the probability that an association persists on the i-th day after it first occurs. Red dashed line denotes the null rate of association calculated based on Whitehead^36^. Periods of no data that occur at yearly intervals correspond to fall-winter (swarming and hibernation period) and no data were collected in 2020. The inset shows the average number of roosts in use by day of the year (Julian Day) across all years of the study where the line represents the smoothed value based on a smoothed loess fit and the shaded area shows the standard error. Fewer roosts used early in each year may correspond to the increased lagged association rate peaks seen at the start of each maternity period.

Lagged association rates across the entire study period declined considerably over the first 60–70 days (1 season), likely related to transient interactions among some dyads with some stable relationships each pregnancy season. This was followed by a slight decline in LAR across years but this metric remained above the null prediction for the entirety of the study period (2012–2021) following the first interaction (Fig. 1). A drop in LAR was seen right before what appears to be the limit of data selection, the expected parturition cutoff, in many years. At the beginning of each season, LAR appeared to spike briefly as bats returned from swarming sites in the first 6 years, but this pattern dissipated beyond this point. This pattern may be related to the fewer number of roosts used on average early in the maternity season causing a greater number of individuals to use each roost box due to social thermoregulatory needs, and thus increasing the probability of association at used roost boxes (Fig. 1 inset).

**Figure 2:**
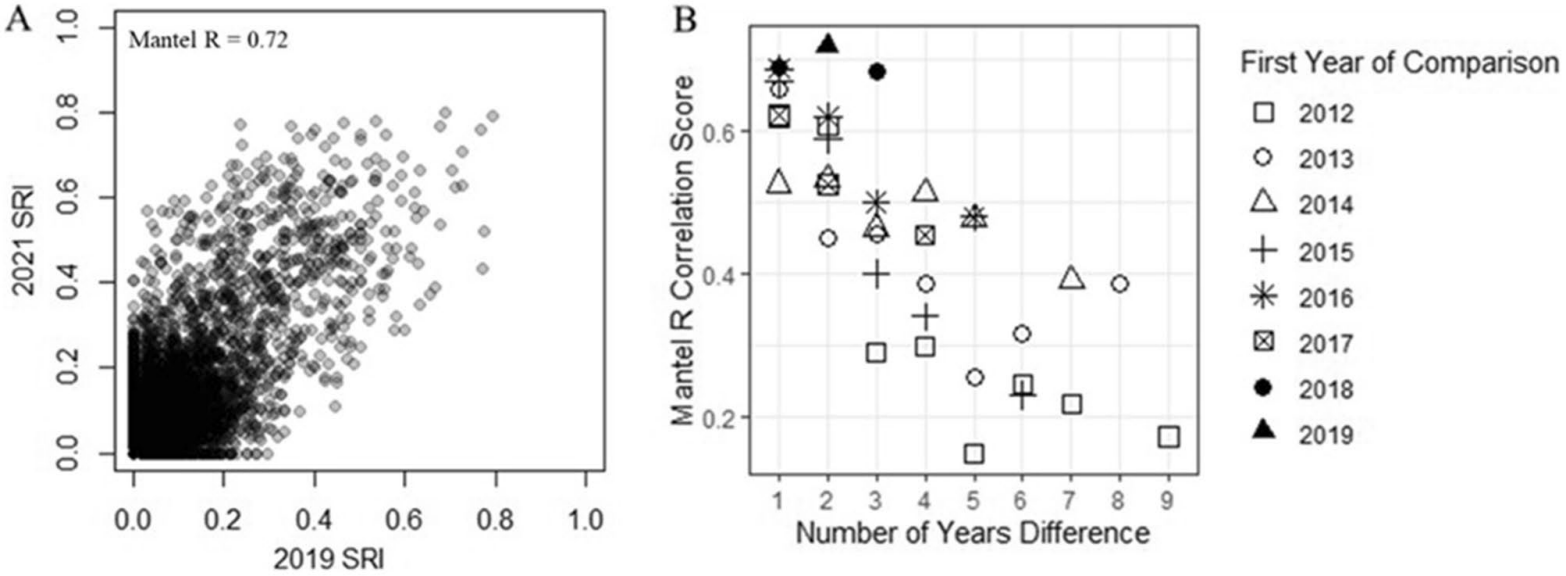
The correlation of association strength (SRI; Simple Ratio Index) between female little brown myotis dyads in Salmonier Nature Park, Newfoundland, Canada, calculated between different combinations of years. (**A**) The SRI in 2019 compared to the SRI in 2021 for all dyads that were present in both years where each point represents one dyad that was present in both years. (**B**) The Mantel R correlation scores for matrices of association strengths in different years, based on the number of years difference between the matrices being compared.

Consistency in association strengths varied greatly between dyads, but overall, networks were significantly correlated (p < 0.05) between years in all comparisons except for association patterns in 2012 compared to association patterns in 2021 (p = 0.40) indicating that relationships between dyads were relatively consistent between years. Mean Mantel R score was 0.46 (SE: 0.03; range: 0.15–0.72). Although it appeared that strong relationships (SRI > 0.5) in one year were predictive of strong relationships in later years based on significant correlations, some relationships did not persist from year to year while other new dyadic relationships appeared among bats that did not co-roost previously (Fig. 2A). Further, whether relationships of moderate strength (SRI 0.2–0.5) became stronger or weaker in subsequent years was highly variable. As predicted, Mantel R scores decreased over time (Fig. 2B).

### Influence of roost fidelity and associate familiarity on network structure

**Figure 3:**
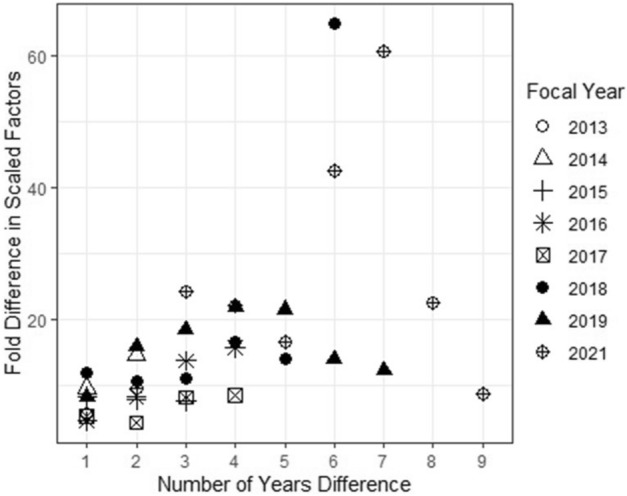
The change in predictive ability of previous year association strengths on co-roosting association networks of little brown myotis in Salmonier Nature Park, Newfoundland, Canada. Fold-difference, calculated as the scaled effect size of focal year probability of roost co-occurrence divided by the scaled effect size of association strengths in the comparison year, was plotted against the number of years difference between the comparison year and the focal year. The fold difference generally increased, indicating a decrease in the predictive ability of comparison year association strengths with a greater amount of time between focal and comparison years. The comparison of 2017 (focal year) to 2012 (comparison year) is not shown as the effect size of the comparison year was 0, and thus a fold difference could not be calculated.

Both the matrix of probability of roost co-occurrence for all individuals in the focal year and the matrix containing association strengths (SRI) between all individuals in the immediately previous year were predictive of the focal year association strengths (p < 0.001). The scaled effect size of the roost co-occurrence matrix was 4- to 65-fold greater than the scaled effect size of the previous year association strengths (Table 1) suggesting a strong predictive ability of roost fidelity. Fold difference could not be calculated for the comparison of 2017 to 2012 as the scaled effect size of the previous year association strengths was zero. The fold difference generally increased with a greater difference between the focal year and comparison year indicating that the predictive ability of previous year association strengths declined when there was more time between the networks being compared (Fig. 3). Association matrices from 2012 were not predictive (p > 0.05) of association matrices in 2016, 2017, 2018, 2019, and 2021 when roost use patterns were accounted for and the association matrices of 2013, 2014, and 2015 were similarly not predictive of association matrices in 2021.

**Table 1:** Scaled effect sizes of the immediately previous year association strengths and roost co-occurrence probability on the association strengths between adult female little brown myotis in the focal year based on a Multiple Regression Quadratic Assignment Procedure (MRQAP). Asterisks indicate signifcant results based on a P value of < 0.05.

Focal year	N	Previous year association		Roost co-occurrence probability		R^2^
		Estimate	p	Estimate	p	
2013	64	0.139	< 0.001*	0.791	< 0.001*	0.778
2014	133	0.086	< 0.001*	0.823	< 0.001*	0.770
2015	132	0.091	< 0.001*	0.755	< 0.001*	0.653
2016	126	0.157	< 0.001*	0.715	< 0.001*	0.692
2017	203	0.144	< 0.001*	0.756	< 0.001*	0.695
2018	151	0.069	< 0.001*	0.814	< 0.001*	0.724
2019	152	0.095	< 0.001*	0.779	< 0.001*	0.727

Estimates of effect size can then be compared to determine which factor was more influential, and to what magnitude, on observed roosting association patterns in the focal year.

### Effect of cohort on association strength

The strengths of associations between bats in the same age cohort were not significantly different from association strengths between bats of different cohorts (t = 0.26, df = 41, p = 0.80). On average, there were 6.4 × as many bats outside of an individual’s cohort than there were from the same cohort within the sample in any given year. Meanwhile, bats on average had 2.5 × as many associates outside of their cohort than within their cohort, within the sample. Some individuals had little to no association with many of the other same cohort individuals despite being present in the system in the same year.

### Influence of age on distribution of social relationships and network position

**Figure 4:**
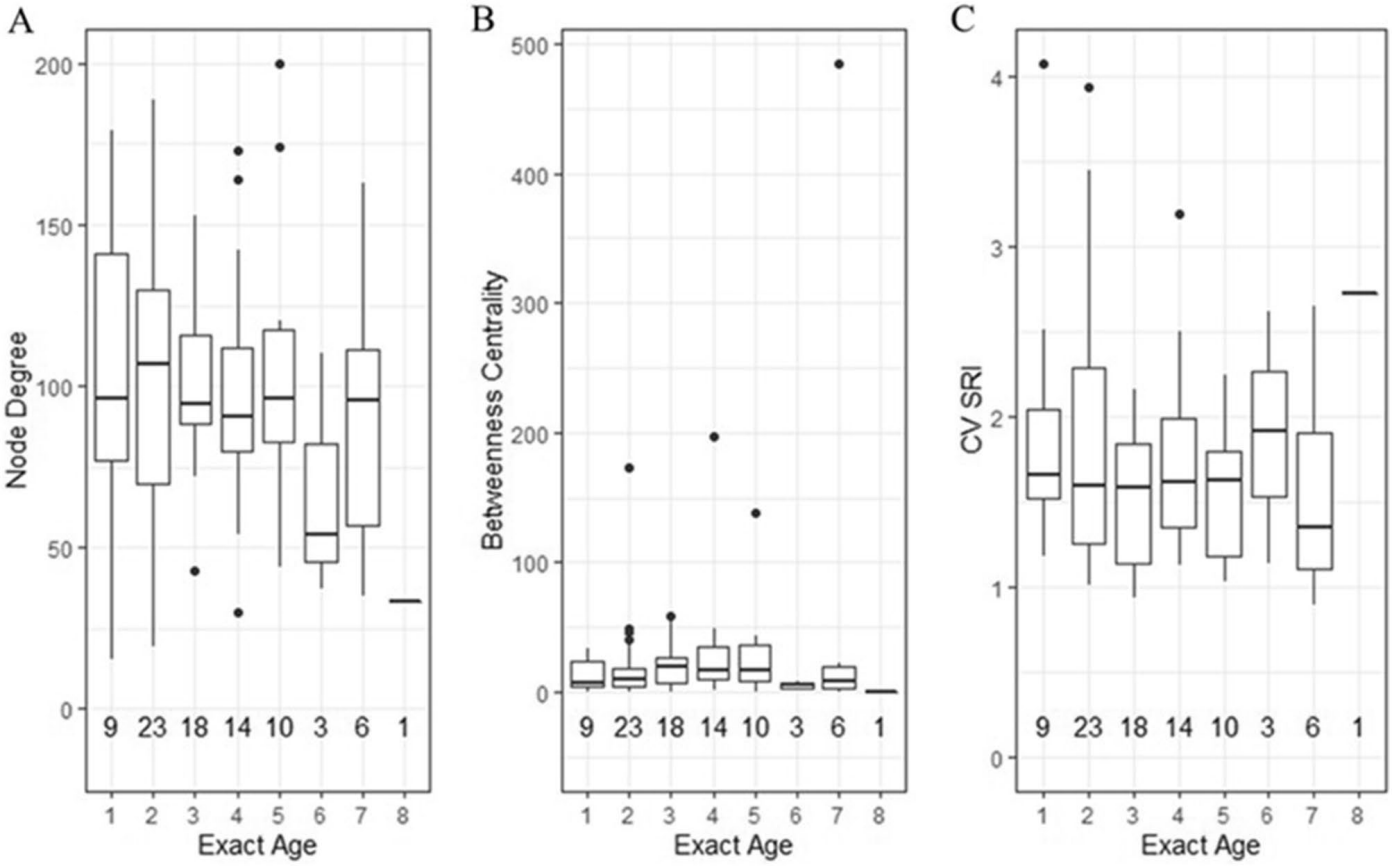
Distribution of network metrics for female little brown myotis in Salmonier Nature Park, Newfoundland, Canada in networks of bats containing at least ten observations at a day roost prior to parturition in a year. Panels show the different individual network metrics of (**A**) node degree, (**B**) betweenness centrality and (**C**) the coefficient of variation of the simple ratio index (CV SRI) at different known ages. Networks were constructed to include individuals of known- and unknown-age, but metrics were only calculated for known-aged individuals. Individuals were assigned an age of 0 in the year they were captured as a juvenile, and thus an exact age of 1 corresponds to the summer a year after birth. The number of known aged individuals for which the metrics could be calculated in each age category are shown along the bottom of each plot.

Individual network metrics did not vary with age in a consistent manner across the known-aged individuals we tested. Node degree was relatively consistent across all ages (Fig. 4A). A slight increase in median betweenness centrality was noted between ages 3 and 5 but may be explained, at least in part, by unequal sample sizes, and therefore might not be biologically significant (Fig. 4B). There was no evidence that there was a difference in CV SRI across the different ages (Fig. 4C). Some individuals were included in multiple exact age categories if they met minimum requirements in multiple years, but the change in network metrics for an individual as they aged was not explored here.

## Discussion

In this study, we failed to reject the hypothesis that bats have persistent social preferences with conspecifics that influence roost-use decisions across years, although the exact cause for the maintenance of relationships over time remains unknown. We found evidence that patterns of roost co-occurrence between adult female little brown myotis persisted over time at a greater rate than what is expected by random chance. We also show that the association strengths between bats in any given year were correlated with association strengths in subsequent years, even when accounting for roost use frequencies. However, the predictive ability of previous year associations was small in comparison to roost use frequencies. By maintaining consistent relationships at maternity sites, bats may accrue benefits such as reduced competition among neighbours and improved information sharing^31^, benefits which may in turn be drivers of long-term relationships. We did not find evidence that, among known-aged individuals, familiarity or experience influenced relationships or network position from year-to-year.,

In mammals, it is common for females to display philopatry and maintain relationships with matrilineal relatives^32^. Although we do not know the relatedness among individuals in this system, or which of the individuals of unknown age are philopatric, it is clear that some bats were returning to this maternity site for at least nine years after we first observed them (including individuals caught at the site as young-of-the-year) and continuing to share roosts with at least some of the same associates. For up to eight years following first observations of shared roost use, the rate of association between many pairs of bats were, on average, greater than expected by chance and association patterns across all dyads were significantly correlated in 35 of 36 year-to-year combinations. We observed that previous year association patterns were still predictive even when accounting for roost fidelity, often for up to a five-year difference between years being compared. Therefore, previous association patterns between bats appear to be indicative of future patterns.

The large difference in scaled effect sizes between factors merits caution, as it is possible that roost use patterns of previous years, and thus multi-year roost fidelity and maternity site philopatry, could be an important factor in multi-year relationships between co-roosting bats. Previous study of little brown myotis in Minnesota showed a mix of philopatric and dispersal behaviours among females^33^ and thus our study may include a mix of philopatric females with individuals that have immigrated from other maternity groups. Other studies of temporal stability in social networks in other species have made similar conclusions, but also encountered similar challenges in teasing apart habitat and social mechanisms^34^. It must be noted that the lagged association rate persisting above the null expectation hypothesis could also indicate fidelity to specific roost boxes within the maternity site, rather than persistent social preferences. In a pipistrelle bat colony (*Pipistrellus pipistrellus*), it was shown that among 17 roost sites, each bat had two to three roosts that it used most frequently^35^ and thus it is possible that little brown myotis in our study are demonstrating similar roost use patterns. However, given that roost use patterns do not entirely explain association patterns for little brown myotis within years^21^, and that even when accounting for roost use patterns, previous year association patterns were predictive of co-roosting relationship between bats, it is reasonable to posit that social preferences could persist across years.

We did not observe a cohort-related or potential cohort familiarity effect, whereby bats in the same age cohort would associate more strongly with each other than with bats of different cohorts. As we apply the gambit-of-the-group assumption here^36^, the assumption that individuals found within the same group are all associating with each other, we cannot be certain that assortment does not occur within roosts as shown previously in other bat maternity groups in buildings^37^. However, when applying the gambit-of-the-group assumption, artificially high assortativity, the tendency to observe individuals associating with those similar to themselves, has been reported when sampling is limited^38^ and so it is equally possible that bats simply do not select associates based on those born in the same year as themselves. Across studies of social networks in bat groups, the role of relatedness in shaping network structure and influencing individual relationships has been mixed, with matriline patterns shown in Spix’s disc winged bats (*Thyroptera tricolor*; Buchalski et al. 2014)^39^. Meanwhile, work on common vampire bats (*Desmodus rotundus*) have shown that familiarity and reciprocity are the main drivers of relationships between bats rather than relatedness^40–42^.

We did not find an effect of age on any of the network metrics observed and thus within the age variation of our dataset, there is no evidence that experience influences an individual’s network position across the range of ages available in our study system to this point. This result contradicts the findings of Patriquin et al.^30^, who noted greater centrality in younger individuals, identified based on the wear of canines in northern long-eared bats (*Myotis septentrionalis*). Keeping in mind the long lifespans of little brown myotis, it is possible that division of adult bats into single-year age classes or the range of ages for which we had sufficient data were not biologically relevant. It is also possible that individual little brown myotis may be specialized to a “social niche” within the larger maternity group, determined by factors such as their individual personality^43^. It is therefore of interest to investigate the relationship between personality and network position, and how network position may change or remain constant across time at the level of individuals.

The degradation of roosts in 2018, and 2021 in our study system have allowed a glimpse into how social relationships may change when critical resources are lost. Mantel R correlation scores and scaled effect sizes in MRQAP appeared similar even in these years where the availability of roosts changed. Temporal heterogeneity in habitat availability can lead to alternate resource selection decisions^11^ and for this reason, it is interesting that association patterns remained consistent in the face of roost availability changes.. These observations make it more difficult to reject the contention that social mechanisms are driving roosting decisions across time and lend greater support for the role of social preference in shaping bat groups across long time periods.

Meanwhile, an understanding of how a change in group composition of individuals changes roost use patterns and therefore network position may further lend support to the role of social preferences in shaping bat groups. White-nose syndrome (WNS), a fungal disease caused by *Pseudogymnoascus destructans* continues to decimate bat populations across North America and is expected to impact the study population in Salmonier Nature Park, Newfoundland, Canada within the next 5 years. If roost use patterns remain the same despite the losses of many individuals, habitat mechanisms may be more influential than social preferences in shaping bat groups. Meanwhile, changes in roost use patterns and consistent relationships despite the loss of many individuals would suggest an important role for social bonds. These effects have been found previously in other species such as flamingos (Phoenicopteridae) where it has been described that long-standing partnerships are important to improve population resilience to environmental fluctuations^44^. Alternatively, strong philopatry or fidelity driving maternity group organization and therefore reduced behavioural flexibility, may reduce the likelihood that this site will be re-colonized by bats from other areas, following a population decline due to WNS, as shown following population stressors to common warthogs (*Phacochoerus africanus*^45^). Understanding the social and habitat selection factors that are influencing maternity group stability will improve our ability to predict how this population will respond to WNS, and what may be needed to assist the recovery of little brown myotis.

Overall, our data supported the contention that roost use patterns and social relationships may be changing slightly year to year, but at least a subset of relationships remain consistent for many years. We provide evidence for long-term relationships in bat maternity groups and our data did not support rejecting the hypothesis that social mechanisms may be responsible for the observed consistency. We also suggest that persistent preferences for specific roosts are an additional contributing factor. Together, by testing the changes and consistencies in association patterns in a bat maternity network, we provide support for the possible presence of persistent social preferences in long-lived temperate bats. Future study should investigate attributes of the individual bats showing consistent relationships, including relatedness and personality measures, to better understand assortment within the maternity group.

## Methods

### Data collection

Little brown myotis were captured in Salmonier Nature Park (Lat: 47.3°, Long: −53.3°) Newfoundland, Canada using mist nets (Avinet, Dryden, New York, USA) and harp traps (Austbat Research Equipment, Lower Plenty, Victoria, Australia) from 2012 to 2021 between 15 May and 19 August of each year. Age^46^, sex, and reproductive status^47^ were recorded for each individual. Bats were marked with passive integrated transponder (PIT) tags (0.09 g; EID-ID100 implantable transponders, EIDAPInc, Sherwood Park, Alberta, Canada and Trovan Electronic Identification Systems, UK) implanted subcutaneously between the scapulae. Transponder antennas (LID650, Dorset Identification, Netherlands) were deployed from April to September each year to collect data on roost entry and exit at eleven roost boxes within a 1.1 km^2^ area. Ten of the eleven roost boxes were positioned in pairs on five poles, and one box was placed on the side of a barn. As many bats were detected in multiple boxes on any one night, it was assumed that the last detection before sunset represented the roost where an individual had spent the day. The last detection before sunset was the same roost as the last detection prior to sunrise the previous morning in 96.9% of all assigned day roost records (> 80,000 records in Salmonier Nature Park), and thus we expect our day roost assignments are reliable. Due to missed detections during PIT-tag monitoring, it is expected that some proportion of roost use was unrecorded. Although not part of the experimental design, due to degradation one roost box was no longer available for bats to use from 2018 onwards. Two other boxes were not monitored during the parturition period in 2018 due to a malfunction but were monitored again in 2019 and 2021. Further, there were three unavailable boxes in 2021 due to degradation, and one available box was not monitored due to malfunction (see Sunga et al.^46^ for additional detail). Data were limited to adult females detected on at least ten days between 28 April and the parturition cut-off in each year as determined by a previous assessment of parturition timing in the same study system^48^. This minimum number of observations was used to ensure that we had a reasonable amount of information on each individual to properly infer their relationships with other included bats and their position in the wider network^49,50^.

All social network and statistical analyses were conducted in R version 4.0.0^51^.

### Social association patterns across years

To quantify the longevity of social relationships based on co-occurrence at a day roost, we computed lagged association rates (LARs), the probability that two individuals re-associate a given number of days from their first observed instance of association^36^. LARs were computed using the function *LAR* in “asnipe”^52^. LAR values were compared to the expected rate if all associations in the network were random (i.e. the null), which was equal to the average number of connections per individual divided by the total number of individuals minus one^36^. We calculated these values based on the gambit-of-the-group assumption^36^, such that bats detected in the same roost on the same day were assumed to be associating through co-roosting.

To assess whether annual roost use and social interaction patterns were consistent among years, we created a matrix of association strengths based on the Simple Ratio Index (SRI) of co-roosting occurrences, whereby we compared the instances of co-roosting to instances of bats roosting separately or when only one bat of a dyad was detected^53^. The SRI was calculated using the function *get_network* in the package “asnipe”^52^. For individual × individual association matrices, we performed Mantel tests for all possible pairwise year combinations to assess the inter-year correlation. Mantel tests were calculated using the function *mantel* in the package “ecodist”^54^. These comparisons included individuals that were present and had at least ten observations prior to parturition in both years, and thus sample sizes varied between comparisons. We then visualized the Mantel test correlation score (R) against the number of years difference between years to investigate whether years closer together in time were generally more similar.

### Influence of persistent social relationships when accounting for roost use patterns

We tested whether the association strength among dyads in previous years was predictive of current association strengths even when roost use patterns were accounted for by using multiple regression quadratic assignment procedure (MRQAP). In this analysis, we regressed the dependent matrix of the individual × individual association strengths in the focal year (2013 to 2021) against the individual × individual association strengths in each previous year representing social influence, and individual × individual co-occurrence probability in the focal year^34^. Co-occurrence probability was based on the probability of overlap at each box based on the product of the % of days each individual spent in each box, summed across all boxes. These analyses for each year were thus limited to individuals present in both the focal and previous years being compared. This approximation of probabilities does not account for days where neither individual was detected as it is possible that individuals still associated at unmonitored roosts or were not recorded in monitored roosts, but we assumed that these observations were missed randomly. The MRQAP procedure was performed using the function *mrqap.dsp* in the package “asnipe”^52^. We then calculated the fold-difference in scaled effect sizes of roost use patterns and previous year association patterns, to investigate whether previous year association strengths were more predictive when compared years were closer together in time.

### Effect of cohort on association strength

For each individual of known age that had at least ten observations prior to parturition in any year (n = 42), an assessment of the number of associates and strengths of associations with other individuals was conducted. To assess the effect of cohort on association patterns, we performed a paired t-test of known aged individuals SRI’s to those of the same cohort, compared to those of different cohorts. This analysis began in 2014 as there were no known aged adults in 2012 and only one known aged adult in 2013. For each individual, we averaged the SRI of within and among cohort dyads across all years to ensure that individuals present in more years or with more associates did not have a greater influence on the analysis than those meeting observation requirements in only a single year. Further, this ensured that the presence of more individuals of different ages than of the same age did not impact the statistical analysis.

### Influence of age on distribution of social relationships and network position

For each individual of known age that had at least ten observations prior to parturition in any year (n = 42) we recorded the coefficient of variation of the simple ratio index (CV SRI), node degree, and betweenness centrality within networks containing all other individuals with at least ten observations prior to parturition in each year, regardless of age status. Although the possible associates could have changed year-to-year, including as many individuals as possible with sufficient numbers of observations should provide the best characterization of the overall network structure and therefore the known-aged individual’s position in it^45^. CV SRI and node degree were selected as we predicted that individuals would become more selective in their associates with age and experience in the system. Thus, the lowest CV SRI values and highest node degree values would be found in the youngest individuals. Node degree, a measure of the number of connections an individual has^55^, was calculated using the function *degree* in the package “igraph” (Csárdi and Nepusz 2006). A measure of centrality was included as it has been shown to vary with age in other social systems^29,30^. Betweenness centrality is a measure of the number of times an individual is a component of the shortest path between two other individuals^55^, and was calculated using the function *centr_betw* in the package “igraph” (Csárdi and Nepusz 2006)^56^. We then performed a linear regression for each of these factors with age as the independent factor.

### Ethics declarations

All animal handling protocol was approved by the animal care committee of Saint Mary’s University, Halifax, Nova Scotia (AUP number 16-12) and the University of Waterloo, Waterloo, Ontario (AUP number 30066). Wildlife scientific research permits were also obtained from the Government of Newfoundland and Labrador, Department of Fisheries and Land Resources, Forestry and Wildlife Branch for each year of the study. All methods were carried out in accordance with relevant guidelines, regulations, and approved animal use protocols and standard operating procedures. All methods are reported in accordance with applicable ARRIVE guidelines.

## Data Availability

The datasets generated during and/or analysed during the current study are not publicly available as data are part of an ongoing, long-term study, but are available from the corresponding author on reasonable request. Example code used for analysis described in this manuscript and to produce the included figures is available on GitHub at https://github.com/juliasunga/roosting_relationships_across_years.
